# Advancements in Sustainable Natural Dyes for Textile Applications: A Review

**DOI:** 10.3390/molecules28165954

**Published:** 2023-08-08

**Authors:** Barbara Pizzicato, Severina Pacifico, Diana Cayuela, Gabriela Mijas, Marta Riba-Moliner

**Affiliations:** 1 Dipartimento di Ingegneria, Università degli Studi della Campania Luigi Vanvitelli, Via Roma 29, 81031 Aversa, Italy; barbara.pizzicato@unicampania.it; 2 Dipartimento di Scienze e Tecnologie Ambientali Biologiche e Farmaceutiche (DISTABiF), Università degli Studi della Campania Luigi Vanvitelli, Via Antonio Vivaldi 43, 81100 Caserta, Italy; severina.pacifico@unicampania.it; 3 School of Industrial, Aeronautical and Audiovisual Engineering of Terrassa (ESEIAAT), Universitat Politècnica de Catalunya, c/Colom 1, 08222 Terrassa, Spain; diana.cayuela@upc.edu (D.C.); gabriela.mijas@upc.edu (G.M.)

**Keywords:** natural dyes, textile, natural dyes extraction, mordanting, biomordants, dyeing process

## Abstract

The dyeing and finishing step represents a clear hotspot in the textile supply chain as the wet processing stages require significant amounts of water, energy, and chemicals. In order to tackle environmental issues, natural dyes are gaining attention from researchers as more sustainable alternatives to synthetic ones. This review discusses the topic of natural dyes, providing a description of their main features and differences compared to synthetic dyes, and encompasses a summary of recent research in the field of natural dyes with specific reference to the following areas of sustainable innovation: extraction techniques, the preparation of substrates, the mordanting process, and the dyeing process. The literature review showed that promising new technologies and techniques have been successfully employed to improve the performance and sustainability of natural dyeing processes, but several limitations such as the poor fastness properties of natural dyes, their low affinity with textiles substrates, difficulties in the reproducibility of shades, as well as other factors such as cost-effectiveness considerations, still prevent industry from adopting natural dyes on a larger scale and will require further research in order to expand their use beyond niche applications.

## 1. Introduction

The global textile industry is responsible for having a serious environmental impact across the entire supply chain, with remarkable greenhouse gas emissions (over 3.3 billion metric tons per year) [1], significant land and water consumption, pollution of the soil, air and water, and increasing waste production.

The current linear system uses large amounts of resources, which creates significant negative impacts on ecosystems and people. It is estimated that every year, about 98 million tons of non-renewable resources are consumed, including, for example, fertilizers to grow natural fibers, oil to produce synthetic fibers, and chemicals used in different stages of textile production [2].

The dyeing and finishing steps represent a clear hotspot in the textile supply chain as the wet processing stages require large volumes of water to be heated and are especially energy intensive. The greenhouse gases emitted from burning fossil fuels to generate the heat and electricity required in these stages of textile production account for their high contribution to the climate impact. According to the UN Environment Programme (UNEP) report on sustainability and circularity in the textile value chain, the contribution of dyeing and finishing to the climate impact of the textile field is accountable for 36% of the entire textile supply chain [3]. The consumption of water is also a major issue, and finishing processes consume great amounts of water, for example to prepare dye baths and wash fabrics after the dyeing process. It is estimated that every kg of cotton requires around 125 L of water to be dyed and finished [4]. Moreover, the dyeing and finishing stage relies heavily on hazardous chemicals and represents a hotspot in terms of carcinogenic human toxicity and a hotspot for non-carcinogenic human toxicity because of the use of detergents, dyes, and water-repellent agents [5].

While promising innovations are emerging to reduce the impact of the dyeing step, such as low water or waterless dyeing processes and chemical-free technologies [6], there is a renewed interest in the topic of natural dyes as a more sustainable alternative to synthetic dyes and a way to reduce the usage of chemicals and the impact on the environment.

This review introduces the topic of natural dyes, providing a description of their main features and differences with their synthetic counterparts and encompasses a summary of recent research in the field of natural dyes with specific reference to the following areas of sustainable innovation: extraction techniques, the preparation of substrates, the mordanting process, and the dyeing process.

### 1.1. Synthetic and Natural Dyes: Advantages and Disadvantages

Synthetic dyes are derived from non-renewable sources, mostly petrochemicals. They have many different complex chemical structures and there are many products in commercial use [7]. It is estimated that globally every year about 800,000 tons of synthetic dyes are produced, and 75% of this amount is consumed by the textile industry [8].

Since their appearance on the market, synthetic dyes have been preferred to natural ones as they are easier to use, they do not require the use of mordants, and they allow to obtain fast, bright, and reproducible shades. However, they can contain insoluble impurities, or generate waste materials that require disposal [9].

Nevertheless, increasing environmental concerns have led researchers to investigate the harmful impacts of synthetic dyes on air, water, and soil. Slama et al. [10] illustrated the effects of toxic gasses released by textile factories such as sulfur derivatives, formaldehyde, oxides of nitrogen, as well as the effects of liquid and solid wastes released into the environment in non-treated or partially treated forms, which especially occurs in those developing countries with insufficient effluent discharge legislation and poor wastewater treatment systems. During the dyeing process, not all the synthetic dye is fixed onto the fabric and the unfixed dye is released into the environment through wastewater, representing a significant amount which is estimated to account for about 10–15% [8]. Usually, the concentration of dye residues in textile wastewater can amount to about 300 mg/L. The presence of dye can be noticed even when its concentration is inferior to 1 mg/L and can have devastating effects on aquatic ecosystems, such as affecting the transparency of water, thus preventing the transmission of light and inhibiting plants’ photosynthesis, altering pH levels, and reducing the quantity of dissolved oxygen [10]. Wastewater from the dyeing process not only contains color but also other unfixed substances such as different auxiliaries, fixing agents, defoamers, oxidizing/reducing agents, and diluents [11]. According to Gürses et al. (2016), typical dye effluents contain high biological oxygen demand (BOD) and chemical oxygen demand (COD) and are abundant in organic and inorganic pollutants such as chlorinated compounds, heavy metals, sulfur, nitrates, naphthol, soaps, chromium compounds, formaldehyde, benzidine, sequestering agents, dyes, and pigments [12].

In addition to environmental impacts, some researchers have pointed out potential effects on human health, both for industry workers and final customers. Exposure to some types of dyes can potentially cause harm to human health, with some of them recognized as respiratory or skin sensitizers [7]. Among these, azo dyes, which were used to dye cotton and are now prohibited by several regulations in the EU, are considered carcinogenic, allergenic, and harmful to the reproductive system. Similarly, disperse dyes, normally used for dyeing synthetic fabrics, are known to have allergenic properties [13]. The European Commission studied the possible correlation between the chemicals used in textile finishing and allergic reactions, reporting that textile finish resins and other textile auxiliaries can induce allergic contact dermatitis [14]. According to research carried out by the Italian Contact and Environmental Dermatitis Research Group, clothing accounts for 8.5% of non-occupational contact dermatitis, with it being the fourth most important cause [15].

**Figure 1:**
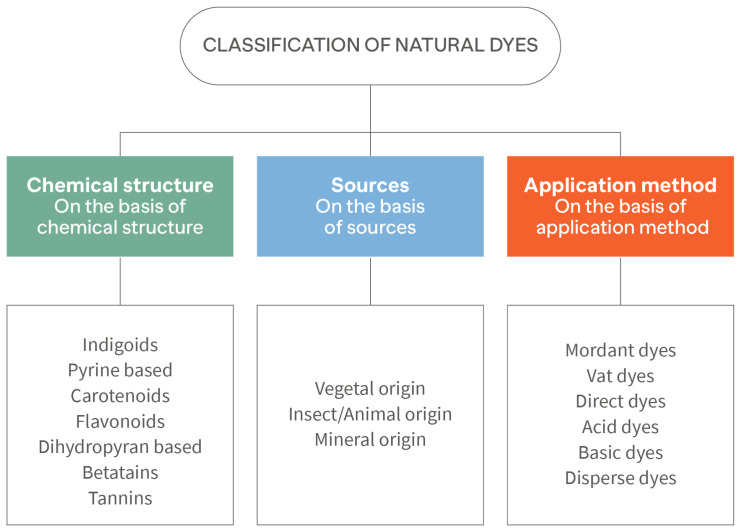
Classification of natural dyes. Adapted from Salauddin et al. [18].

Natural dyes, on the other hand, are considered eco-friendly as they are sourced from renewable resources and are biodegradable. Natural dyes sources are generally classified into plants (e.g., indigo and madder), minerals (e.g., ochre and clay), animals (e.g., cochineal and some species of Mollusca) [16], and microbes, although plants are the most common source. Various parts of the plant can be used, such as the roots, leaves, twigs, stems, heartwood, bark, and wood [17]. In Figure 1, a classification of natural dyes is provided [18].

Moreover, research has shown that natural dyes may lend several functional properties to textiles, such as antibacterial, antifungal, UV protective, insect repellent, and aromatic properties, due to a group of active biomolecules known as phytochemicals, which differ based on the plant considered as well as their mechanism of action. Kamboj et al. listed the major classes of microorganisms causing degradation to textiles, analyzed the key factors affecting the antimicrobial activity of natural dyed fabrics, reported the main phytochemicals responsible for the antimicrobial activity of natural dyes—such as saponins, tannins, flavonoids, glycosides, and anthocyanins—and their mode of action [19].

For instance, pomegranate (*Punica granatum*) is reported to have significant antimicrobial properties thanks to its high concentration of tannins [20]. In one study, cotton dyed with *Butea monosperma*, marigold, banana pseudostem sap, and pomegranate rind extracts showed remarkable antibacterial activity against the two microorganisms considered by researchers, namely *S. aureus* and *E. coli* [21]. Hwang and Hong [22] examined viscose rayon dyed with the extract of Aleppo oak (*Quercus infectoria*), assessing its excellent antioxidant properties.

Baseri [23] investigated the UV protection properties of bio-cotton dyed with waste pomegranate rind, finding that the final fabric exhibited excellent protection against ultraviolet radiation (UPF 50+). Similarly, Hou et al. [24] compared the UV protection factor (UPF) of wool dyed with orange peel extracts through direct dyeing and the same value for wool dyed with synthetic dyes and similar shade and depth of shade, concluding that the UPF of naturally dyed wool was about six times higher than the latter.

Despite the clear environmental advantages of using natural dyes, several authors have pointed out many technical issues and disadvantages which still prevent the industry from adopting them on a larger scale. A major source of concern for the industry is the poor fastness of natural dyes. Natural dyes show low dye uptake, poor fastness, and uneven dyeing. Mordanting substances—often metallic salts, which are not always environmentally sustainable—are needed to improve the fastness and to fix the dye to the fabric [18].

The type of bonding between dyes and fibers directly affects the fastness properties of dyed textiles. Synthetic fibers do not show affinity with natural dyes, which can only be used to dye natural fibers. Nevertheless, among the same natural fibers, protein fibers are easy to dye with natural dyes due to the presence of ionic groups in their structure, while cellulosic fibers are more difficult to dye as there is a lack of bonding between natural dyes and the fiber. Due to their poor substantivity, it is necessary to pretreat cellulosic fibers through a mordanting process [25].

Another critical issue is the reproducibility of shades, as the results obtained are related to numerous factors, such as the features of the natural dye matter, maturity, variety, agroclimatic conditions, soil type, and weather. Some natural dyes are pH sensitive; the difference in the mineral content and pH of the water used could also affect the results of the dyeing process. Therefore, it is impossible to achieve standardized and consistent results [17] although the uniqueness of the shades obtained could be considered as an added value for niche applications.

Moreover, the dyeing process with natural dyes is long and expensive and requires a considerable quantity of water and a significant amount of raw material to achieve the same depth of color as synthetic dyes [24]. Repeating dye batches to achieve the same shades implies significant consumption of water, dyestuff, and energy every time the process is repeated.

### 1.2. Natural Dyes Sourcing

The availability of dye matter is also a matter of concern, depending on several factors such as seasonality. The available supply of natural dyes is just 1% of world demand (10.000 tons) [26]. Moreover, the consumption of land to grow raw materials to extract the dye and the use of pesticides on the crop may also affect the sustainability of the final fabric [27].

A more sustainable sourcing option—both in terms of environmental and economic sustainability—is represented by the usage of agro-industrial waste as renewable raw material for natural dye production. Phan et al. [28] explored the potential of natural dyes extracted from food waste by analyzing the amount of fruit and vegetables waste and by-products derived from agricultural losses and industrial processing in Europe and providing mapping based on the concentration of anthocyanins, quinones, and carotenoids in the waste and by-products examined. The authors considered several technological and methodological aspects, describing possible routes of valorization and concluding that food waste streams can be considered a competitive source to obtain natural dyes, especially in terms of niche applications.

Recently, studies on agro-industrial waste as a source to dye different textile substrates are multiplying, demonstrating both the environmental and economic benefits of adopting a circular approach [29,30,31,32]. Obtaining dyes from waste and by-products would be a preferable option in terms of production costs if compared to direct harvesting. Nevertheless, obtaining the same depth of color as primary agriculture dye matter with waste is still a challenge [33].

The application of natural dyes on a large industrial scale will require the overcoming of numerous technical challenges, such as the improvement of extraction and application methods in order to increase their cost-effectiveness [34]. The advantages and disadvantages of natural dyes are summarized in Table 1.

## 2. Advancements in Natural Dyes Extraction

The extraction of colorant from natural sources is a fundamental step in preparing purified natural dyes, as a plant’s matrix contains only a small percentage of dye, usually in the range of 0.5–5%, and several other constituents such as water-insoluble fibers, carbohydrates, protein, chlorophyll, and tannins, among others. The selection of the most suitable extraction technique should be based on the evaluation of the nature and solubility of the dyeing materials [17].

### 2.1. Aqueous Extraction

Aqueous extraction is a traditional method in which dye matter is usually first reduced to small pieces or powdered and then immersed in water to loosen the cell structure and improve the efficiency of the process. The dye solution is obtained by boiling and then filtered. The extraction and filtration process can be repeated several times [35].

Aqueous extraction is a sustainable and safe technique, and the extract can be easily applied to textiles. The disadvantages are its long extraction time, the large amounts of water required, and the low dye yield as only the water-soluble dye components are extracted. Moreover, sugars and other water-soluble components are extracted along with the dye. Yields of heat-sensitive dyes are reduced at high temperatures [17].

Pervaiz et al. [36] performed a simple extraction in aqueous medium to obtain dye through the valorization of marigold (*Tagetes erecta* L.) waste flowers by soaking petals for several hours, boiling them, leaving the solution to cool, and then filtering it through filter paper. Different extraction conditions were compared, and it was reported that the maximum extraction yield was obtained when extraction was performed at 40 °C for 40 min.

### 2.2. Solvent Extraction

Similar to aqueous extraction is extraction with organic solvents such as ethanol or methanol or a mix of solvents, which allows for a higher extraction yield, however. Lower temperatures can be used, limiting chances of degradation. Water/alcohol extraction can extract both water-soluble and water-insoluble components. Moreover, the solvents can be easily removed through distillation to be reused. Disadvantages include the presence of toxic residual solvents. Additionally, the extracted material is not readily soluble in water; the co-extraction of other substances such as chlorophylls and waxy materials could occur [17].

Al-Alwani et al. [37] compared the effectiveness of nine solvents, namely n-hexane, ethanol, acetonitrile, chloroform, ethyl ether, ethyl acetate, petroleum ether, n-butyl alcohol, and methanol to extract natural dyes from cordyline, pandan, and dragon fruit (respectively *Cordyline fruticosa, Pandannus amaryllifolius,* and *Hylocereus polyrhizus*), assessing the best extraction conditions. The results obtained showed that the most suitable solvents for the dye extraction of the plants considered were methanol, ethanol, and water.

### 2.3. Alkali or Acid Extraction

Similar to the previous techniques, extraction under alkali or acid conditions can facilitate the hydrolysis of glycosides with higher extraction yields—as many dyes are in the form of glycosides. Alkaline extraction is particularly suitable for dyes containing phenolic groups, which are soluble in alkali conditions. A disadvantage of this extraction technique is that alkaline conditions could damage the dyeing matter, as many dyes are pH sensitive [25].

Alkaline extraction of natural dye from grape pomace, a by-product of wine production, was performed using sodium hydroxide and compared to aqueous extraction. The results showed that a higher extraction yield was obtained through alkaline extraction. Moreover, different extraction conditions (sodium hydroxide concentration, grape pomace amount, extraction duration, and temperature) were studied to optimize the dyeing process of wool fabric with extracted dye [38].

### 2.4. Ultrasound- and Microwave-Assisted Extraction

In ultrasound-assisted extraction (UAE) and microwave-assisted extraction (MAE) the dye matter is treated with water or other solvents in the presence of ultrasounds or microwaves. These processes provide a better extraction yield, lower extraction temperatures, lower solvent usage, and less time and energy consumed. The possibility of using lower temperatures is more suitable for heat-sensitive molecules [39].

Ultrasounds are defined as mechanical waves characterized by a frequency above 20 kHz (human hearing range). The waves can propagate in solids, liquids, and gasses through compression and rarefaction cycles. When high intensity waves propagate in a liquid medium, the negative pressure during the rarefaction phase is stronger than the force attracting molecules together, causing molecules’ dispersion and the formation of cavitation bubbles. These bubbles grow until they collapse, generating the phenomenon known as cavitation, with an increase in temperature and pressure. Cavitation is an important mechanism exploited in the ultrasound-assisted extraction of bioactive compounds. As a matter of fact, the collapsing of bubbles causes a series of mechanisms such as erosion or pore formations, which can ultimately facilitate the breaking of plant matrix cells and the release and solubilization of compounds of interest [40].

Several authors have used ultrasound-assisted extraction to study the potentiality of new dye sources, investigating the best process conditions [41,42,43] for application on different textile substrates—natural, regenerated, and synthetic substrates.

Wizi et al. [44] individuated sorghum husk (an agro-industrial by-product) as a promising source of natural dyes for textiles due to the high amounts of phenolic colorants which are tightly bound to the cell walls, however, and therefore difficult to extract. Ultrasound technology was employed in combination with microwaves to increase the extraction efficiency. Moreover, the effects of different solvents were investigated. The extracts were subsequently used to dye wool and cotton. The results showed that a higher color strength was obtained when the extraction was performed with 70:30 ethanol:water mixture (*v*/*v*) with HCl. The dyed wool and cotton fabrics showed good fastness values to washing, crocking, and light. Additionally, it was noticed that extracts with different solvents led to different shades on wool and cotton fabrics.

Microwaves are included in the electromagnetic spectrum and are characterized by wavelengths ranging between 0.001 and 1 m and frequencies ranging between 0.3 and 300 GHz. Compared to conventional heating, heating through microwaves shows higher efficiency (up to 50%), resulting in significantly inferior energy consumption [45].

Unlike traditional heating techniques in which heat is transferred from the equipment to the solution, microwaves allow for direct heating of the solution, resulting in a faster process with a lower temperature gradient. Moreover, microwave-assisted extraction allow for the significantly lower consumption of organic solvents [46].

In the microwave-assisted extraction (MAE) of plant metabolites, the main mechanism induced by microwaves is the generation of heating and dipole rotation in organic molecules of the plant matrix. This leads to an increase in kinetic energy and friction between the ions, eventually causing the breaking of hydrogen bonding, as well as facilitating the penetration of the solvent in the vegetal matrix [47], resulting in a significant reduction in organic solvent consumption.

A better extraction yield was obtained through the microwave-assisted extraction of cinnamon bark as dye matter for biomordanted silk fabric. It was observed that by using an aqueous medium, microwave extraction enhanced the color strength (K/S). The type of medium greatly affected the results.

Chemat et al. [48] discussed the potential of ultrasound-assisted extraction in combination with microwave-assisted extraction (MAE), as microwave irradiation provides fast and efficient extraction but inhomogeneous heating. Combination with ultrasounds was presented as a solution to overcome this issue.

### 2.5. Enzymatic Extraction and Fermentation

Extraction through enzymes is considered an environmentally friendly technique to extract active compounds from plant matrices, avoiding the use of solvents. Enzymes act as catalysts and are used to extract, modify, and synthetize natural active compounds [49].

Appropriate enzymes such as cellulase, amylase, and pectinase are used to decompose plant tissues under mild conditions, helping the release of active compounds and increasing the speed of extraction. Temperature and pH are the main factors that affect the activity of enzymes [17]. These techniques are particularly suitable for hard plant materials such as the bark and roots [24].

Tiwari et al. [50] compared enzymatic extraction with pectinase and cellulase, ultrasound extraction and enzyme-assisted extraction, and the enzyme-mediated ultrasonic-assisted extraction of natural colorants from pomegranate rind. The dyeing behavior of te extracted dyes on cotton and wool was also investigated, and it was found that the combination of enzymes and ultrasounds gave the highest results in terms of color yield.

### 2.6. Supercritical Fluids

During the last two decades, supercritical fluids have gained popularity in the extraction of organic compounds from plant matrices due to several advantages [51].

Supercritical fluids are defined as substances above their critical pressure and temperature which possess properties of both liquids and gasses. The critical values depend on the specific substance. When a gas is above its critical temperature and pressure, it is compressed in a supercritical fluid and is characterized by a density similar to that of a liquid, a viscosity similar to that of a gas, and a diffusion coefficient between liquids and gasses. Thanks to these properties, supercritical fluids possess high solvating power and diffusivity and low viscosity and surface tension. These characteristics allow for fast mass transfer in supercritical fluids. Consequently, in extraction with supercritical fluids (SFE) the mechanism of penetration of the solvent into the matrix is facilitated, resulting in a fast and efficient extraction process. Another advantage of supercritical fluids is that the changing pressure, temperature, and density affect the solubility of these substances [52]. Additionally, the density of the fluid can be modified by altering the pressure and temperature values. Therefore, in the extraction process with supercritical fluids, the solvent’s strength can be also regulated by modifying the various parameters. Supercritical fluids’ extraction comprises two main steps, which are the solubilization of the extract in the supercritical solvent and the consequent separation of the extract from the solvent. In the first stage, the absorption of the supercritical solvent by the plant matrix causes the swelling of its cellular membranes and a decrease in mass transfer resistance. At the same time, the extracted compounds are solubilized and move to the external surface of the cell. Afterwards, the solubilized compounds are transported from the surface of the cell to the solvent. Finally, in the last stage, they are removed from the supercritical solvent [53,54].

Several solvents can be used as supercritical fluids, such as carbon dioxide, ethane, ethene, methanol, nitrous oxide, n-butene, n-pentane, sulfur hexafluoride, and water, although carbon dioxide (CO_2_) is the most commonly used, and it is estimated that more than 90% of all supercritical fluid extractions (SFEs) are carried out with CO_2_. This is due to several reasons: firstly, carbon dioxide is non-toxic and non-flammable, and it is considered safe for human health and the environment in terms of manipulation and between certain value ranges. Secondly, it is available at high purity, at relatively low cost, and is easy to remove from the extract. Moreover, it has a low critical pressure and temperature (73 atm and 31.2 °C, respectively), factors that favor the preservation of the bioactive compounds contained in the extracts [53,55].

The main disadvantage of the use of CO_2_ as a supercritical solvent is its low polarity, which makes it an efficient solvent for the extraction of compounds with no or low polarity but ineffective at extracting polar compounds. Nevertheless, the addition of small amounts of so-called modifiers—polar organic solvents, such as methanol—has been proven an adequate strategy to improve the extraction efficiency of CO_2_, amplifying its extraction range with the inclusion of more polar compounds [52,56].

In order to perform selective extraction of the desired compounds only, the complex interplay between thermodynamics (solubility compounds to be extracted or of the undesired compounds) and kinetics (mass transfer resistance) must be considered. In this context, microscopic analysis is a useful tool that allows for the identification of the mass transfer resistance in the structure of the matrix [57].

Overall, supercritical fluid extraction presents many advantages: it is an efficient process in terms of high yields and low extraction times, requires low or room temperatures, and uses solvents generally recognized as safe (GRAS); the extract does not contain residual solvent; and it is possible to directly couple the extraction process with analytical chromatographic techniques such as gas chromatography (GC) or supercritical fluid chromatography (SFC) [58,59].

The main disadvantages include the high cost of the technology, the inefficient extraction of polar substances [17], and risks to workers. As a matter of fact, systems that use fluids in supercritical conditions operate at very high pressure, which is much higher than atmospheric pressure. This aspect represents a potential danger for the workers involved in the process and requires the adoption of preventive safety measures and risk analysis [60].

**Figure 2:**
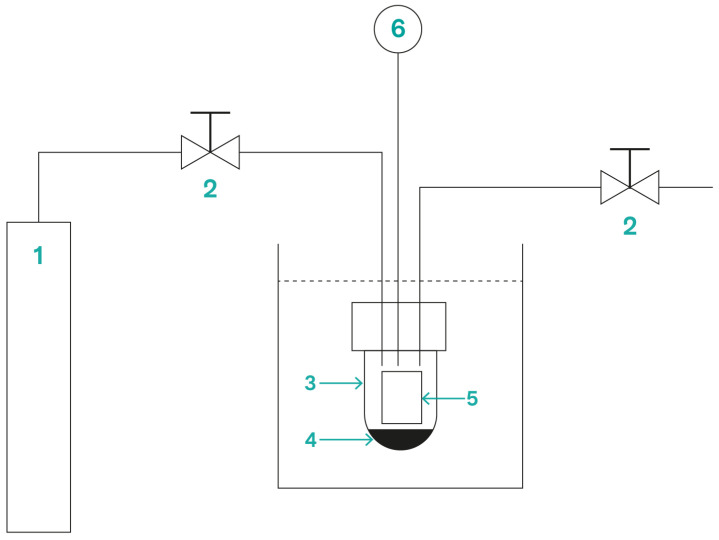
Dyeing of the PET fabric with supercritical CO_2_. Note: 1—CO_2_ cylinder, 2—valve, 3—reactor vessel, 4—turmeric, 5—fabric, and 6—pressure gauge. Adapted from Kabir et al. [61].

Kabir et al. [61] used supercritical fluids to carry out an innovative dyeing process for PET fabric with curcuminoid dyes from turmeric, in which the simultaneous extraction of natural dyes and the dyeing process itself were carried out in the same supercritical bath using carbon dioxide. As for the parameters used, the temperature, pressure, and time were respectively set at 150 °C, 20 MPa, and 1 h. The resulting samples were compared with samples obtained through conventional dyeing with ethanol-extracted dyestuff, and it was found that the PET samples dyed with a supercritical carbon dioxide technique were characterized by the highest dye exhaustion, K/S, and fastness properties. Figure 2 schematically represents the process of the extraction and dyeing of PET through the use of supercritical CO_2_.

## 3. Advancements in Mordanting and Substrate Pretreatment

Prior to the dyeing step, the fabrics must be adequately prepared through a pretreatment process aimed at removing all natural impurities or residual impurities from the previous production steps and improving the water absorbency, dyeability, and whiteness of the fabric. The pretreatment process adopted depends on the type of fabric and can involve complex steps. In the case of cotton, pretreatment includes desizing, scouring, and bleaching [62].

Poor affinity with textile fabrics, poor color strength (K/S), and the fastness properties of natural dyes represent obstacles to their adoption on an industrial scale [17]. Natural dyes are most effective on natural fibers. The two major categories of natural fibers are protein fibers, such as wool and silk, and cellulosic fibers, such as cotton. Both have an affinity with natural dyes, although protein fibers are easier to dye than cellulosic fibers. As a matter of fact, within the molecular structure of protein fibers are amino groups (alkaline), carboxyl groups (acid), and OH groups. The presence of both amino and carboxyl groups ensures that the fabric will react with both acids and bases. For this reason, protein fibers form strong bonds with mordants, which in turn bond with the dyes. Cellulose, on the other hand, contains primarily OH groups that cannot bind to most dyes or mordants, so cellulosic fibers require a different approach to mordanting and dyeing [63]. Due to the ionization of carboxyl and hydroxyl groups, cellulosic fibers possess a slightly negative charge when immersed in water. At the same time, most natural dyes are negatively charged. Consequently, when dyeing cellulosic fibers with natural dyes, the electrostatic repulsion between the dye and the anionic structure of the cellulosic fibers represents the main cause of low dye uptake and poor color strength [64].

Therefore, to improve the substantivity of natural dyes, fabrics undergo pretreatment processes such as treatment with mordants and biomordants. In addition, recently, new, eco-friendly techniques have emerged to increase the dyeability of textiles with the application of natural dyes. These include enzyme treatment; irradiation treatments such as plasma, ultrasonic, microwave, ultraviolet, and gamma radiation; and nanotechnology. These processes can modify the surface of the material at the nanometer scale without affecting its bulk properties, avoiding the usage of hazardous chemicals and drastically reducing the amount of water needed [65].

### 3.1. Mordants and Biomordants

**Figure 3:**
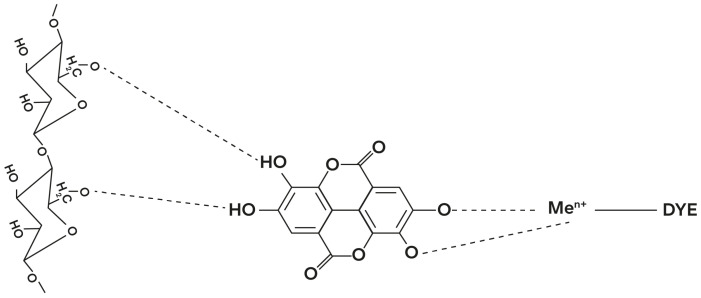
Mechanism of cotton with tannins, metallic mordants, and dye. Adapted from Prabhu and Buthe [67].

To overcome the poor substantivity and fastness properties of natural dyes, fabrics are traditionally treated with substances called mordants, used to form a stable dye–metal complex and fix the color to the fabric. The word “mordant” is derived from the French verb *mordre* which means “to bite”. The mechanism of action is different depending on the type of fiber; in the case of protein fibers, the mordant binds to it, while in cellulose fibers, it is left as an insoluble compound on the fiber and the dye binds to the mordant (Figure 3) [66,67].

Mordants are usually metal salts (e.g., aluminum, iron, copper, tin, and chrome) used to fix a dye to a fabric; however, the most used mordants are a variety of chemical salts made from aluminum; iron salts are often used as a post-dye treatment to darken colors; and copper is used mainly as a post-mordant, but sometimes as a pre-mordant and requires care in handling and disposal [63]. With the same dyestuff, different types of mordant generate different dye complexes and different results for color coordinates, color strength, and fastness values. Moreover, the moment in which the mordanting process takes place directly affects the results. This can be carried out prior to the dyeing process (pre-mordanting), at the same moment of the dyeing process (simultaneous/meta-mordanting), or as an extra step after the dyeing process (post-mordanting) [68].

Traditional mordants used in natural dyeing have often been associated with risks to the environment, due to the presence of residuals in the wastewater after the dyeing process [69,70]. As a more environmentally friendly alternative to conventional metallic mordants, recent research has been exploring the use of biomordants, obtained from natural sources. In Figure 3, a classification of mordants is provided [68].

A wide range of plants and biomaterials can replace the use of metallic mordants in the fixation of natural dyes onto fabrics, with their effectiveness strongly depending on their chemical structure and content. Biomordants include a wide range of substances, such as tannins, tannic acid, and tartaric acid. For example, several plants have been successfully used as biomordants due to their high content of tannins and chlorophyll [71]. Some plants known as hyperaccumulators (e.g., tea, camelia, club moss, and symplocos) are naturally capable of absorbing aluminum through their roots and can be used as biomordants [63]. Moreover, biomordants can also be obtained from biowastes and by-products [68].

Like metallic mordants, the type and concentration of biomordant employed influences the color yield and the fastness properties of the dyed fabric and in some cases can lead to unsatisfactory results. As a matter of fact, a multitude of factors can affect color coordinates, color strength, and fastness, such as the natural dye source, the type and the concentration of mordant, the mordanting technique, the extraction technique, and the parameters of the dyeing process [72].

For example, the effect of metal mordants and bio-mordants (tannic acid, pinecone, and lemon peel) on cotton dyed with *Hibiscus sabdariffa* L. was compared. The biomordants were found useful in increasing the color fastness of the dyed textiles and obtaining good values of light fastness, wash fastness, and dry/wet rub fastness, as well as showing antibacterial activity (>90% bacterial reduction) [73].

Similarly, Hosen et al. [74] compared the effects of biomordants derived from lemon and taro (*Citrus limon* and *Colocasia esculenta,* respectively) to those of metallic mordants (potassium dichromate and potash alum) in the pre-mordanting of cotton dyed with turmeric (*Curcuma longa* L.) extract, obtaining a two-times higher color-strength for the biomordant pretreated sample. The color fastness to rubbing (dry and wet), washing, and perspiration were also investigated, and better results were found for bio-crosslinkers compared to metallic salt chelation.

On the other hand, the use of black wattle (*Acacia mearnsii*) as a biomordant on banana fiber was found satisfactory. Compared with the alum-mordanted fibers, the black wattle biomordanted fibers resulted in a more intense coloration, with no changes in the appearance and softness of the fabric [75].

Rani et al. [76] studied the dyeing process of wool with papaya (*Carica papaya* L.) leaf as a natural dye and biomordants. The dye was extracted and reduced to dry powder. As for the mordant used, different metal salts (ferrous sulfate, alum, and copper chloride) were tested and compared to natural tannin extracts as biomordants (harda powder, pomegranate peel, orange peel, and amla powder). Additionally, the process was performed with and without mordants and compared all mordanting techniques (pre-mordanting, meta-mordanting, and post-mordanting). Afterward, the results were evaluated in terms of color strength and fastness properties, showing that the effects of the bio-mordants were comparable with those of conventional metallic mordants.

Some research has been carried out on the possible application of natural dyes in combination with biomordants on synthetic fibers as well. The effect of several bio-mordants, including peppermint (*Mentha piperita*), mugworts (*Artemisia*), gum ammoniac (*Dorema ammoniacum*), and pomegranate rind, was considered in the dyeing process of nylon fabric (polyamide 6) with dragon’s blood extract—a resin which is obtained from *Calamus* spp. The color strength values were found to be acceptable in comparison to samples treated with metallic mordants [77].

Recently, several studies have focused on the possibility of employing biomordants obtained from agro-industrial waste and by-products, such as orange peel, pomegranate rind, or banana peel [78,79,80].

### 3.2. Enzyme Treatment

Enzymes have been used in numerous textile processes such as desizing, scouring, bleaching, dyeing, and finishing as a more sustainable alternative to the currently used chemicals [81]. However, it is estimated that only about 75 enzymes are used in textile industry processes within the 7000 known [82]. For example, enzymes such as lipase, pectinase, xylanase and cellulase are commonly used in textile processes of pretreatment and finishing [83] in order to modify the physical and chemical properties of the fiber surface or introduce specific functional groups onto it [84]. Other examples include amylases, which are employed in desizing; cellulases, commonly used in denim finishing and the biopolishing of cellulosic fibers; proteases, used in leather, silk, and wool treatment; and pectinases—amylase, lipase, and diasterase—used in the biopreparation of cotton fabrics [85].

The use of enzymes to improve the natural dyeing of fibers—especially cellulosic fibers—is a promising area of research, although more efforts are needed to select the appropriate enzymes and study their compatibility with natural dyes for large-scale applications [86]. Samant [87] studied the pretreatment of cotton by comparing conventional and enzymatic techniques. Three enzymes were employed (acid cellulase, neutral cellulase, and xylanase) to pretreat cotton, which was dyed afterward with *A. catechu* dye. It was found that all enzyme treatments improved the dyeing efficiency, and the color fastness values as well as enhancing the ultraviolet protection, and antimicrobial efficacy of the cotton fabric.

Benli and Bahtiyari [88] studied the use of an enzymes–ultrasounds combination in the same bath to treat cotton before the dyeing process with natural dyes. Different dyes obtained from pomegranate peel, nutshell, orange tree leaf, and alkanet root were compared. In order to better understand the effect of the enzymes, no mordants were used. The results showed that the treatment helped to remove non-cellulosic matter from undyed cotton and improve the desizing, hydrophilicity, and whiteness effect. Additionally, the dyed samples showed higher color efficiency and darker shades.

Raja and Thilagavathi [89]’s wool pretreated with the alkaline protease enzyme and subsequently dyed with four different natural dyes was found to have significantly higher color difference (ΔE) and color strength than the non-treated samples.

Similarly, Zhang and Cai [90] evaluated the effect of protease and transglutaminase in the pretreatment of wool dyed with sappan. Although the enzymatic treatments had no influence on the washing fastness, treatment with protease and transglutaminase was found to be useful in improving the color strength values of dyed wool.

### 3.3. Irradiation Technologies

Irradiation technologies such as ultrasound radiation, ultraviolet radiation, gamma radiation, electron beam irradiation, and plasma treatment are gaining popularity for use in textile dyeing and finishing processes as they represent a more sustainable alternative to the conventional wet processes.

**Figure 4:**
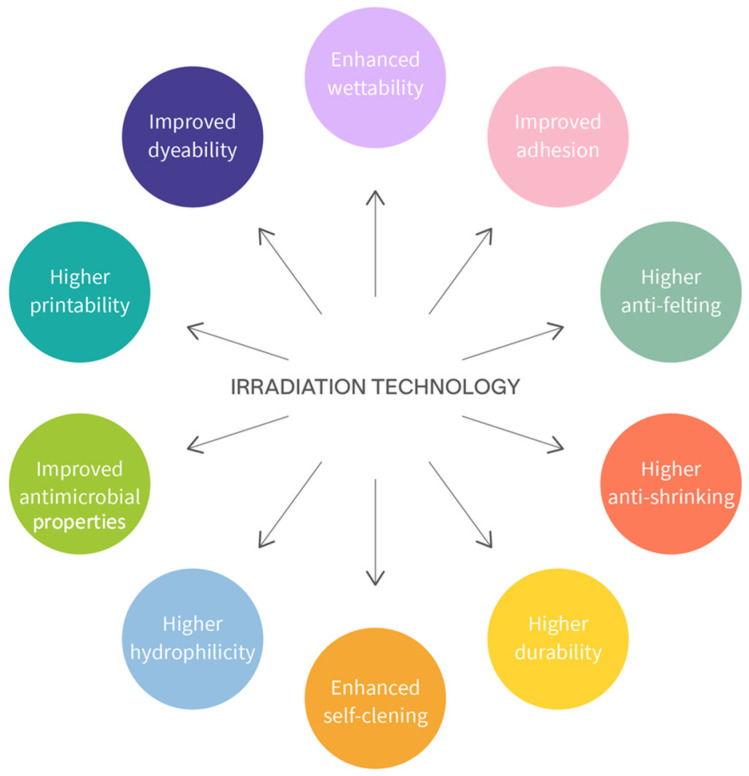
Possible effects of irradiation technologies on textile materials. Adapted from Islam and Mohammad [91].

In fact, these innovative technologies are essentially fast, low-temperature processes, which require low amounts of water and chemicals. Irradiation technologies are used in dyeing and finishing to modify the textile surface and impart different qualities and functional properties without affecting the bulk properties of the material. For example, they are used to improve adhesion, dyeability, fastness properties, resistance to wrinkling, and susceptibility to microbial attacks. In Figure 4, various effects of irradiation technologies on textile materials are shown [91].

In the field of natural dyes, these techniques have been successfully employed to overcome the poor affinity of natural dyes and enhance dye uptake, fastness properties, and the overall dyeability of the fabric [65,92]

Ultrasound technology is reported to be beneficial in numerous studies on natural dyes and in different stages of the natural dye process to enhance the extraction of natural dyes, substrate pretreatment, and dyeing process. In terms of fabric pretreatment, research shows its effectiveness in removing dirt and contaminants from natural fibers such as grease, wax, vegetable matter, and pigments as a more energy-efficient, time-saving and inexpensive alternative to the methods currently available [91].

Moholkar et al. [93] noticed that ultrasounds generate an important effect of enhancement of mass transfer, useful in processes of the pretreatment and finishing of fabrics, as limited mass transport in the inter-yarn and intra-yarn pores is the main cause making wet processes highly time and energy consuming. Moreover, a cleaning effect is registered, due to the oscillations of acoustic bubbles and micro-jets produced from the collapse of acoustic bubbles, which in turn generate convective mass transfer in the intra-yarn pores.

Several authors have successfully employed ultrasounds to improve the efficiency of dirt removal and fabric preparation in natural fibers, in processes such as scouring, desizing, bleaching, and mercerizing [94,95,96].

Kadam et al. [97] studied the application of ultrasounds in the scouring wool and reported that ultrasound energy was effective in removing grease, allowing for the lower consumption of chemicals and decreasing the temperature and duration of the process without affecting the quality of the fabric.

Ultraviolet radiation is included in the electromagnetic spectrum and is characterized by a wavelength range of 100–400 nm. This technology is often used in fabric pretreatment and presents the advantage of affecting only the chemical properties of the upper layers of the material without changing the bulk properties, thus avoiding mechanical loss. In the preparation of fabrics for the dyeing process, ultraviolet treatment was found effective in enhancing dye uptake, producing deeper shades and improving the fastness properties without affecting the bulk properties of the material [91,98].

Ultraviolet radiation (UV) is often used in cotton pretreatment as it introduces carboxylic groups onto the fabric surface which enhances interaction between the fabric and the dye molecules [99].

In two different studies, Adeel et al. [100] and Guzar et al. [101] studied the application of ultraviolet treatment to improve the natural dye of cotton, exposing both the fabric and dye powder to UV radiation. In both cases, UV treatment was found effective. In the dyeing process with turmeric (*Curcuma longa* L.) in powder form, better values of color strength and color fastness of the dyed samples were obtained thanks to the application of UV, with a lower concentration of dye and mordants. In the dyeing of cotton with barks of kikar (*Acacia nilotica*) as a source of natural flavone dye (quercetin), it was observed that UV radiation improved the results of color fastness tests from poor to good.

Similarly, Rehman et al. [102] studied the dyeing behavior and properties of UV-irradiated cotton with the application of marigold as a source of natural Lutein dye. UV radiation was used to pretreat both the dye and the fabric before the dyeing process, which was carried out by testing different parameters. The optimum conditions for surface modification and dyeing were assessed and it was concluded that UV ray treatment can be used in enhancing the natural dye process without any physical damage.

Gamma irradiation, or gamma rays, is an electromagnetic radiation of high frequency that is produced by subatomic particle interactions. Gamma rays have the smallest wavelengths (from 10^−10^ to 10^−13^ m) and the most energy (from 10^19^ to 10^24^ Hz) compared to any other wave in the electromagnetic spectrum [103].

In textile substrates’ treatment, this technology provides an economical, fast, and efficient process, which requires low temperatures and energy consumption and no use of chemicals. In the context of the dyeing process, it can improve dye uptake, color strength and color fastness [91,104,105]. Nevertheless, gamma irradiation requires careful handling by workers, and it may affect the mechanical properties of the fiber. As observed by many researchers [106,107,108], in the treatment of cotton, high amounts can damage the fabric, causing the chemical degradation of cellulose, with it ultimately becoming more rigid and more friable and losing its mechanical properties due to a depolymerization effect and a decrease in crystallinity.

However, several studies have shown the efficacy of gamma-ray treatment on the improvement of dyeing processes with natural dyes. Cotton treated with gamma radiation and successively mordanted and dyed with red calico leaves extract showed improved color fastness properties. Different doses of gamma radiation were tested and resulted in differences in the color strength values. A dose of 15 kGy was found to be the optimum dose for cotton modification, as the hydroxyl group of cellulose units was transformed into a carboxylic group, allowing their interaction with the dye molecules [109].

Similarly, Gulzar et al. [101] applied acacia (*Acacia* sp.) bark extract to gamma-irradiated cotton. The authors exposed both cotton fabric and dye to gamma rays and tested different doses and dyeing variables, finding that 20 kGy was the most effective dose for cotton pretreatment. Moreover, abundant hydroxyl group one C-6 and two C-2, C-3 of the cellulosic group favored extensive hydrogen bonding, which led to good values of color strength and fastness.

Electron beam irradiation (EBI) consists of a flow of energetic electrons generated by an accelerator, which can modify the chemical and physical properties of materials.

Accelerated electrons interact with polymeric materials through a process that involves physical, physicochemical, and chemical transformations. In the first stage, the electrons gradually transfer their energy to the polymeric material. This generates reactive species such as excited states, ions, and secondary electrons, which are then converted into polymer radicals. The two main mechanisms activated by EBI on polymeric materials are the formation of chemical bonding between polymer chains (cross-linking) and the degradation of polymer chains. EBI is similar to gamma radiation, although it varies in dose rates and penetration and presents additional advantages such as higher efficiency due to the directivity of the electron beam and easier management and maintenance of the radiation source. Moreover, it is an environmentally friendly, solvent-free, and fast process [110,111,112].

Elmaaty et al. [112] collected several studies on the EBI treatment of textile materials to enhance the properties of the fabric in the context of dyeing of different substrates (natural, synthetic, and regenerated fibers), and observed that many researchers found this technology effective in improving wettability, dyeability, color strength, and color fastness.

**Figure 5:**
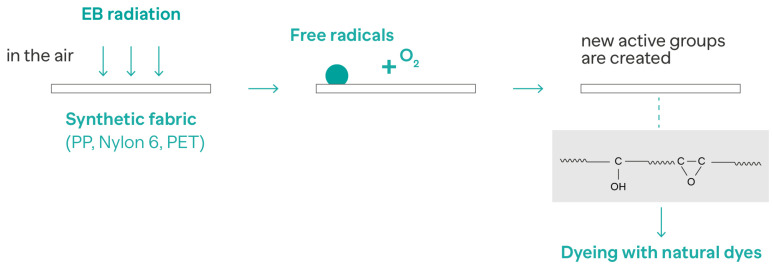
The dyeing of synthetic fabrics pretreated with EBI. Adapted from Elmaaty et al. [113].

In a previous study, Elmaaty et al. [113] used EBI pretreatment on different synthetic fabrics, namely polypropylene (PP), polyamide 6, and polyethylene terephthalate (PET), to enhance dyeability with natural dyes (saffron and turmeric) (Figure 5). The variables studied included different exposure doses and oxidation periods in order to find the optimum conditions. The samples treated were analyzed and showed excellent durability and color fastness for roughness, rubbing, and washing.

Another irradiation technology is plasma, which is defined as a partially ionized gas constituted by many types of species, such as positive and negative ions, electrons, neutrals, excited molecules, photons, and UV light [114].

Numerous types of plasma have been used to treat all kinds of fibers (natural, synthetic and regenerated), using a variety of gasses and gas mixtures depending on the specific application, even though the most common plasma treatments are helium/oxygen or helium/air. The main functions of plasma applied to textiles can be summarized in modifying the chemical structure as well as the surface properties of textiles [115]; depositing substances (plasma polymerization) [116] to increase functionality; and removing materials (plasma etching) [117] from textiles for better applicability [65,118].

Plasma treatment is a fast and dry process which does not require solvents or water and requires no or very low amounts of chemicals. Therefore, the advantages of plasma include the reduction of water consumption, chemicals, energy, time, and waste produced. It introduces new functional properties and improves color fastness without affecting the bulk properties. A potential drawback is the high cost of some of the gases that can be used, specifically helium [91].

Haji et al. [119] investigated the effect of low-temperature oxygen plasma for the pretreatment of cotton before dyeing it with madder and weld extracts. It was found that plasma treatment greatly improved the dyeability of cotton and fastness properties with both natural dyes with and without mordant. Plasma affected the surface layer of cotton, generating an etching effect and removing impurities; at the same time, it allowed for the creation of carbonyl groups on the fabric surface so as to increase the dye uptake and the fastness properties of the treated samples.

Similarly, oxygen plasma was employed to improve the dyeability of wool fibers with an aqueous extract of grape leaves. The samples obtained were examined through an electron microscopy scan, and it was found that the effect of plasma was useful in removing scales on the wool surface, thus improving the penetration of natural dye [120].

## 4. Advancements in the Dyeing Process

Dyeing is defined as the aqueous application of color to fiber, yarn, or fabric. In addition to dyes, an industrial dyeing process requires the use of other auxiliary chemicals in order to obtain uniform colorations and acceptable fastness properties of the final product. Since dyeing is a wet process which involves the significant consumption of water and energy, research is constantly focused on developing efficient, more environmentally sustainable processes. To this extent, new technologies such as ultrasonic, microwave, plasma, supercritical carbon dioxide coloration, and nanotechnology have been employed to enhance the dyeing process [121].

The dyeing process with natural dyes is usually carried out by the exhaustion method, as industrial machinery is not optimized to work with natural dyes, with the most important parameters being the material-to-liquor ratio (MLR), temperature, time, and pH [17].

Although many studies in the dyeing process focus on synthetic dyes, recent advancements in natural dyes show attempts to apply new technologies to bring innovation to this area of research.

Ultrasounds have been employed to enhance several chemical and physical processes, thanks to the cavitation phenomenon. Improvements have been observed in ultrasound-assisted coloration processes, and numerous researchers have applied this technique to textile dyeing on different substrates [122,123,124]. Some studies have reported the use of ultrasound-assisted dye with natural dyes. Vankar et al. [85] studied the dyeing of cotton using *Eclipta alba* L. as a natural dye, comparing both conventional and sonicator methods. The latter method resulted in higher color strength values and better dye uptake. The fastness properties of the dyed fabrics were found to be satisfactory.

In microwave-assisted dyeing, energy is absorbed directly by the dye molecules, inducing their oscillation which in turn favors the diffusion of dye in the fiber structure, unlike conventional heating methods in which heat is gradually transferred [125]. In one study, the dyeing of polyester fabric was performed using microwave energy and henna natural dye, and it was reported that the fixation (%), the rate of dye uptake, and the dyeing time were reduced up to 60–65% as compared to conventional heating [126].

Another technique that can be considered is the use of supercritical carbon dioxide (CO_2_) as a solvent instead of water, allowing for a more environmentally friendly waterless dyeing process, as well as easier separation of the residual dye from the solvent [127].

Many studies have highlighted the advantages of using supercritical CO_2_ in the dyeing process, especially for polyethylene terephthalate (PET) dyeing [128,129,130] and with dispersed dyes. The effectiveness of the supercritical fluid dyeing of PET with disperse dyes lies both in the fact that disperse dyes display higher solubility in supercritical CO_2_ than in water and that CO_2_ molecules can easily penetrate and swell the empty space between the polymer chains of PET, increasing their mobility through a phenomenon known as plasticization and therefore allowing for better diffusion of the dye. Since the first pilot-scale development of the process by UHDE GmbH in 1995, many other prototypes have been developed. In the last few years, companies such as DyeCoo and Hisaka have started producing and selling machinery for the dyeing of polyester in supercritical CO_2_. The Dutch company DyeCoo reports that each of their machines can process 800,000 kg of PET per year, with a significant saving of water (32 million L) and a drastic reduction in the consumption of chemicals (160,000 kg saved). Furthermore, about 95% of CO_2_ can be recovered and reused after each process [131,132].

Research on nanotechnology has shown the possibility of using pigment nanoparticles in textile processes, including the dyeing process. In this respect, difficulties in the application of pigment nanoparticles are obtaining particles of adequately small size and the tendency of nanoparticles to aggregate in the dyebath [122]. Ragheb et al. [133,134] developed a process for the printing natural fabrics with natural dyes, curcuma, and pomegranate peel, respectively, via nanotechnology. In both cases, the nanoparticles were developed through an ultrasonic stirrer.

Ionic liquids are a relatively new group of solvents that have gained the interest of researchers in the last 20 years, with them finding applications in different fields of green chemistry thanks to their properties [135], but still, their use remains limited due to high costs and the lack of scientific information. Ionic liquids (ILs) are normally defined as compounds completely composed of ions with a melting point below 100 °C [136]. Ionic liquids possess several advantages, with one of the main ones being their modularity and the possibility to tailor them according to one’s needs; their physicochemical properties such as their density, viscosity, and solubility in water are influenced by the structure of the cations and anions. By varying the combination of cations and anions or changing their structure, it is possible to obtain the most suitable compound for the application desired. Moreover, ionic liquids are also considered as environmentally friendly as they can be successfully recycled and reused in other processes [132].

Various authors have employed ionic liquids such as deep eutectic solvents for the extraction of bioactive compounds and pigments [137,138,139]. Mekto and Nomngongo [140] reviewed the use of ionic liquids in order to extract phenolic compounds and dyes.

In the field of textiles, ionic liquids are employed, for instance, in textile spinning for the dissolution of cellulose in the production of fibers of regenerated cellulose; in textile preparation to increase the dyeability of fabrics; and in textile dyeing, finishing, and wastewater treatment [132]. In textile dyeing, ionic liquids were used as auxiliaries in the dye bath [141] and in the replacement of water in an innovative waterless dyeing technique first developed by Deutsche Textilforschungszentrum Nord-West (DTNW) [142,143].

## 5. Conclusions and Further Research

This review focuses on the topic of natural dyes as an alternative to synthetic ones and a means to address the negative environmental impact of textile dyeing and finishing wet processes. The main advantages and disadvantages of natural dyes were discussed, as well as issues related to their sourcing.

A literature review was carried out concerning recent advancements in the field of natural dyes with reference to extraction techniques, the mordanting process and substrate preparation, and natural dyes’ application, and several studies investigating innovative technologies and techniques in the field of natural dyes were reported.

As regards to sourcing, interesting opportunities of sustainable innovation are generated by the employment of agro-industrial waste and by-products as natural dyes sources, which avoids the land consumption linked to direct harvesting and does not compete with other applications such as food.

As for the extraction of natural dyes, innovative techniques, such as ultrasound- and microwave-assisted extraction or enzymatic extraction are already being employed to reduce the consumption of water, energy, and chemicals characterizing the traditional extraction methods. In this context, the potentiality of supercritical fluid extraction is still not fully exploited in the field of natural dyes but indeed could allow water-less and more sustainable extraction, helping to reduce the great amounts of water needed to extract natural dyes.

As concerns textile substrate pretreatment, advancements in the use of biomordants and enzymes could soon replace metallic salts as mordants, while new promising technologies have been investigated such as ultrasound radiation, ultraviolet radiation, gamma radiation, electron beam irradiation, and plasma treatment. These irradiation technologies could help overcome the poor fastness of natural dyes through a sustainable process, as they usually consist of fast, low-temperature processes, requiring low amounts of water and chemicals.

Finally, in the dyeing process, encouraging advancements have been made thanks to the application of nanotechnology, plasma, ultrasound, microwave, and new disruptive techniques such as supercritical carbon dioxide coloration. Little information is available about the dyeing process with ionic liquids applied to natural dyes, which could represent a promising alternative, however, drastically reducing the amount of water used in the process.

Although the literature review demonstrated great interest in the topic of natural dyes, limitations such as poor fastness properties, low affinity with textiles substrates, especially with synthetic fabrics, difficulties in the reproducibility of shades, as well as other factors such as cost-effectiveness considerations or difficulties in adapting natural dyes to the pre-existing industrial systems, still prevent natural dyes from being adopted on an industrial scale. To overcome these limitations and allow for natural dyes’ application to be expanded beyond niche applications, a concerted effort in all the above-mentioned areas of innovation will be necessary.

## Figures and Tables

**Table 1:** Natural dyes: advantages and disadvantages.

Advantages	Disadvantages
Extracted from renewable sources Biodegradable and environmentally friendly Possess functional properties (e.g., antibacterial, antifungal, UV protective, insect repellent, and aromatic) Unique shades are obtained Waste and by-products can be used as a source (e.g., wine and avocado peels)	Poor dye uptake, color strength, and fastness Poor affinity with many textile fibers and no affinity with synthetic fibers Require mordants Non reproducibility of standard shades Long, expensive, water-consuming process

## Data Availability

Data sharing not applicable. No new data were created or analyzed in this study. Data sharing is not applicable to this article.

## Sample Availability

Not available.

## References

[1] Quantis (2018). Measuring Fashion: Insights from the Environmental Impact of the Global Apparel and Footwear Industries. Full Report and Methodological Considerations. https://quantis.com/wp-content/uploads/2019/11/measuringfashion_globalimpactstudy_quantis_2018.pdf.

[2] Ellen MacArthur Foundation (2017). A New Textiles Economy: Redesigning Fashion’s Future. https://ellenmacarthurfoundation.org/a-new-textiles-economy.

[3] UN Environment Programme (UNEP) (2020). Sustainability and Circularity in the Textile Value Chain.

[4] Turley D.B., Horne M., Blackburn R.S., Stott E., Laybourn S.R., Copeland J.E., Harwood J. (2009). The Role and Business Case for Existing and Emerging Fibres in Sustainable Clothing: Final Report to the Department for Environment.

[5] Sandin G., Roos S., Peters G. (2019). Environmental Assessment of Swedish Clothing Consumption—Six Garments, Sustainable Futures. https://core.ac.uk/download/pdf/270109142.pdf.

[6] Maxwell D.M., Andrew L., Ryan J. (2015). The State of the Apparel Sector 2015 Special Report—Water a Report for the Global Leadership Award in Sustainable Apparel.

[7] Health and Safety Executive (HSE) (2016). Dyes and Chemicals in Textile Finishing: An introduction. Dyeing and Finishing Information Sheet No 1—HSE Information Sheet. https://www.hse.gov.uk/textiles/dyes-dyeing.htm.

[8] Hassaan M.A., Nemr A.E. (2017). Health and Environmental Impacts of Dyes: Mini Review. Am. J. Environ. Sci. Eng..

[9] Bide M. (2014). Sustainable dyeing with synthetic dyes. Roadmap to Sustainable Textiles and Clothing: Eco-Friendly Raw Materials, Technologies, and Processing Methods.

[10] Slama H.B., Chenari Bouket A., Pourhassan Z., Alenezi F.N., Silini A., Cherif-Silini H., Oszako T., Luptakova L., Golinska P., Belbahri L. (2021). Diversity of Synthetic Dyes from Textile Industries, Discharge Impacts and Treatment Methods. Appl. Sci..

[11] European Commission Zero Brine, D6.1 Wastewater and Solution Provider Knowledge Models, Correlations and Interlinks, October 2020. https://ec.europa.eu/research/participants/documents/downloadPublic?documentIds=080166e5d4a61977&appId=PPGMS.

[12] Gürses A., Açıkyıldız M., Güne¸s K., Gürses M.S., Gürses A., Açıkyıldız M., Güne¸s K., Gürses M.S. (2016). Classification of Dye and Pigments. Springer Briefs in Molecular Science.

[13] KEMI, Swedish Chemicals Agency (2016). Hazardous Chemical Substances in Textiles, Proposals for Risk Management Measures.

[14] European Commission Final Report, Study on the Link Between Allergic Reactions and Chemicals in Textile Products, 7 January 2013. https://commission.europa.eu/.

[15] Lisi P., Stngeni L. (2010). Gruppo Italiano Ricerca Dermatiti da Contatto e Ambientali (GIRDCA) epidemiological survey of contact dermatitis. Am. J. Contact Dermat..

[16] Affat S.S. (2021). Classifications, advantages, disadvantages, toxicity effects of natural and synthetic dyes: A review. Univ. Thi-Qar J. Sci..

[17] Saxena S., Raja A.S.M. (2014). Natural dyes: Sources, chemistry, application and sustainability issues. Roadmap to Sustainable Textiles and Clothing: Eco-Friendly Raw Materials, Technologies, and Processing Methods.

[18] Salauddin S., Mia R., Haque M.A., Shamim A.M. (2021). Review on extraction and application of natural dyes. Text. Leather Rev..

[19] Kamboj A., Jose S., Singh A. (2022). Antimicrobial activity of natural dyes—A comprehensive review. J. Nat. Fibers.

[20] Kannahi M. (2013). Vinotha, Antimicrobial activity of Lawsonia inermis leaf extracts against some human pathogens. Int. J. Curr. Microbiol. Appl. Sci..

[21] Iqbal S., Ansari T.N. (2021). Extraction and application of natural dyes. Sustainable Practices in the Textile Industry.

[22] Hwang H.J., Hong K.H. (2016). Effect of pretreatment on Dyeability and functionalities of summer rayon fabrics finished by gallnut extract. Fash. Text. Res. J..

[23] Baseri S. (2020). Eco-friendly production of anti-UV and antibacterial cotton fabrics via waste products. Cellulose.

[24] Hou X., Chen X., Cheng Y., Xu H., Chen L., Yang Y. (2013). Dyeing and UV-protection properties of water extracts from orange peel. J. Clean. Prod..

[25] Gupta V.K. (2019). Fundamentals of natural dyes and its application on textile substrates. Chemistry and Technology of Natural and Synthetic Dyes and Pigments.

[26] Senthilkumar R., Vaneshwari V., Sathiyavimal S., Amsaveni R., Kalaiselvi M., Malayaman V. (2015). Natural Colors from dyeing plants for textiles. Int. J. Biosci. Nanosci..

[27] Khattab T.A., Abdelrahman M.S., Rehan M. (2020). Textile dyeing industry: Environmental impacts and remediation. Environ. Sci. Pollut. Res..

[28] Phan K., Raes K., Van Speybroeck V., Roosen M., De Clerck K., De Meester S. (2021). Non-food applications of natural dyes extracted from agro-food residues: A critical review. J. Clean. Prod..

[29] Moussa I., Ghezal I., Sakli F. (2023). Valorization of Pelargonium graveolens L’Hér. Hydrodistillation Solid Waste as Natural Dye for Wool Fabrics. J. Nat. Fibers.

[30] Zhang Y., Zhou Q., Rather L.J., Li Q. (2021). Agricultural waste of *Eriobotrya japonica* L.(Loquat) seeds and flora leaves as source of natural dye and bio-mordant for coloration and bio-functional finishing of wool textile. Ind. Crops Prod..

[31] Sukemi P.K., Srisuwannaket C., Niamnont N., Mingvanish W. (2019). Dyeing of cotton with the natural dye extracted from waste leaves of green tea. Color. Technol..

[32] Jose S., Pandit P., Pandey R. (2019). Chickpea husk—A potential agro waste for coloration and functional finishing of textiles. Ind. Crops Prod..

[33] Ganglberger E. (2009). Environmental aspects and sustainability. Handbook of Natural Colorants.

[34] Shahidi S., Khoshechin E., Sharifi S.D., Mongkholrattanasit R. (2022). Investigation of the Effect of Various Natural Dyes on UV Protection Properties and Antibacterial Activity of Cotton Fabrics. J. Nat. Fibers.

[35] Merdan N., Seyda E., Mujgan N.D. (2017). Ecological and sustainable natural dyes. Textiles and Clothing Sustainability: Sustainable Textile Chemical Processes.

[36] Pervaiz S., Mughal T.A., Khan F.Z., Hayat S., Aslam A., Shah S.F. (2017). Environmental friendly leather dyeing using *Tagetes erecta* L. (Marigold) waste flowers. Int. J. Biosci..

[37] Al-Alwani M.A., Mohamad A.B., Kadhum AA H., Ludin N.A. (2014). Effect of solvents on extraction and adsorption of natural dyes extracted from *Cordyline fruticosa* and *Hylocereus polyrhizus*. Asian J. Chem..

[38] Baaka N., Ticha M.B., Haddar W., Hammami S., Mhenni M.F. (2015). Extraction of natural dye from waste wine industry: Optimization survey based on a central composite design method. Fibers Polym..

[39] Adeel S., Habib N., Arif S., ur Rehman F., Azeem M., Batool F., Amin N. (2020). Microwave-assisted eco-dyeing of bio mordanted silk fabric using cinnamon bark (*Cinnamomum verum*) based yellow natural dye. Sustain. Chem. Pharm..

[40] Kumar M., Dahuja A., Tiwari S., Punia S., Tak Y., Amarowicz R., Kaur C. (2021). Recent trends in extraction of plant bioactives using green technologies: A review. Food Chem..

[41] Ma X., Wei Y., Wang S., Zuo X., Shen B. (2020). Sustainable ultrasound-assisted ultralow liquor ratio dyeing of cotton fabric with natural turmeric dye. Text. Res. J..

[42] Sadeghi-Kiakhani M., Hashemi E., Gharanjig K. (2019). Inorganic nanoparticles and natural dyes for production of antimicrobial and antioxidant wool fiber. 3 Biotech.

[43] Qadariyah L., Mahfud M., Sulistiawati E., Swastika P. (2018). Natural dye extraction from teak leves (Tectona Grandis) using ultrasound assisted extraction method for dyeing on cotton fabric. MATEC Web of Conferences.

[44] Wizi J., Wang L., Hou X., Tao Y., Ma B., Yang Y. (2018). Ultrasound-microwave assisted extraction of natural colorants from sorghum husk with different solvents. Ind. Crops Prod..

[45] Tylor M., Atri B.S., Minhas S. (2005). Development in Microwave Chemistry.

[46] Chaturvedi A.K. (2018). Extraction of Nutraceuticals from Plants by Microwave Assisted Extraction. Syst. Rev. Pharm..

[47] Akhtar I., Javad S., Yousaf Z., Iqbal S., Jabeen K. (2019). Microwave assisted extraction of phytochemicals: An efficient and modern approach for botanicals and pharmaceuticals. Pak. J. Pharm. Sci..

[48] Chemat F., Rombaut N., Sicaire A.G., Meullemiestre A., Fabiano-Tixier A.S., AbertVian M. (2017). Ultrasound assisted extraction of food and natural products. Mechanisms, techniques, combinations, protocols and applications. A review. Ultrason. Sonochem..

[49] Gardossi L., Poulsen P.B., Ballesteros A., Hult K., Švedas V.K., Vasić-Rački Đ., Carrea G., Magnusson A., Schmid A., Wohlgemuth R. (2010). Guidelines for reporting of biocatalytic reactions. Trends Biotechnol..

[50] Tiwari H.C., Singh P., Kumar Mishra P., Srivastava P. (2010). Evaluation of various techniques for extraction of natural colorants from pomegranate rind—Ultrasound and enzyme assisted extraction. Indian J. Fibre Text. Res..

[51] Pourmortazavi S.M., Hajimirsadeghi S.S. (2007). Supercritical fluid extraction in plant essential and volatile oil analysis. J. Chromatogr. A.

[52] Mitra S. (2003). Sample Preparation Techniques in Analytical Chemistry.

[53] Da Silva R.P., Rocha-Santos T.A., Duarte A.C. (2016). Supercritical fluid extraction of bioactive compounds. TrAC Trends Anal. Chem..

[54] Brunner G. (1994). Supercritical gases as solvents: Phase equilibria. Gas Extraction: An Introduction to Fundamentals of Supercritical Fluids and the Application to Separation Processes.

[55] Elmaaty T.A., Abd El-Aziz E. (2018). Supercritical carbon dioxide as a green media in textile dyeing: A review. Text. Res. J..

[56] Björklund E., Sparr-Eskilsson C. (2005). Extraction, Supercritical Fluid Extraction. Encyclopedia of Analytical Science.

[57] Reverchon E., De Marco I. (2016). Supercritical fluid extraction and fractionation of natural matter. J. Supercrit. Fluids.

[58] Borges M.E., Tejera R.L., Díaz L., Esparza P., Ibáñez E. (2012). Natural dyes extraction from cochineal (*Dactylopius coccus*). New extraction methods. Food Chem..

[59] Herrero M., Cifuentes A., Ibañez E. (2006). Sub-and supercritical fluid extraction of functional ingredients from different natural sources: Plants, food-by-products, algae and microalgae: A review. Food Chem..

[60] Iovine A., Leone G.P., Larocca V., Di Sanzo G., Casella P., Marino T., Musmarra D., Molino A. (2020). Risk analysis of a supercritical fluid extraction plant using a safety software. Chem. Eng..

[61] Kabir SM M., Hasan M.M., Uddin M.Z. (2019). Novel approach to dye polyethylene terephthalate (PET) fabric in supercritical carbon dioxide with natural curcuminoid dyes. Fibres Text. East. Eur..

[62] Vankar P.S. (2017). Natural Dyes for Textiles: Sources, Chemistry and Applications.

[63] Boutrop J., Ellis C. (2018). The Art and Science of Natural Dyes: Principles, Experiments, and Results.

[64] Pisitsak P., Hutakamol J., Jeenapak S., Wanmanee P., Nuammaiphum J., Thongcharoen R. (2016). Natural dyeing of cotton with Xylocarpus granatum bark extract: Dyeing, fastness, and ultraviolet protection properties. Fibers Polym..

[65] Singh M., Vajpayee M., Ledwani L. (2021). Eco-friendly surface modification of natural fibres to improve dye uptake using natural dyes and application of natural dyes in fabric finishing: A review. Mater. Today Proc..

[66] Broadbent A.D. (2001). Basic Principles of Textile Coloration.

[67] Prabhu K.H., Bhute A.S. (2012). Plant based natural dyes and mordants: A Review. J. Nat. Prod. Plant Resour..

[68] İşmal Ö.E., Yıldırım L. (2019). Metal mordants and biomordants. The Impact and Prospects of Green Chemistry for Textile Technology.

[69] Rahman N.A., Tajuddin R., Tumin S. (2013). Optimization of natural dyeing using ultrasonic method and biomordant. Int. J. Chem. Eng. Appl..

[70] Rovira J., Nadal M., Schuhmacher M., Domingo J.L. (2015). Human exposure to trace elements through the skin by direct contact with clothing: Risk assessment. Env. Res..

[71] Guesmi A., Hamadi N.B., Ladhari N., Sakli F. (2012). Dyeing properties and color fastness of wool dyed with indicaxanthin natural dye. Ind. Crop Prod..

[72] İşmal Ö.E. (2017). Greener natural dyeing pathway using a by-product of olive oil; prina and biomordants. Fibers Polym..

[73] Shahmoradi Ghaheh F., Moghaddam M.K., Tehrani M. (2021). Comparison of the effect of metal mordants and bio-mordants on the colorimetric and antibacterial properties of natural dyes on cotton fabric. Color. Technol..

[74] Hosen M.D., Rabbi M.F., Raihan M.A., Al Mamun M.A. (2021). Effect of turmeric dye and biomordants on knitted cotton fabric coloration: A promising alternative to metallic mordanting. Clean. Eng. Technol..

[75] Pinheiro L., Kohan L., Duarte L.O., Garavello M.E.D.P.E., Baruque-Ramos J. (2019). Biomordants and new alternatives to the sustainable natural fiber dyeings. SN Appl. Sci..

[76] Rani N., Jajpura L., Butola B.S. (2020). Ecological dyeing of protein fabrics with Carica papaya L. leaf natural extract in the presence of bio-mordants as an alternative copartner to metal mordants. J. Inst. Eng. Ser. E.

[77] Haji A., Shahmoradi Ghaheh F., Mohammadi L. (2023). Dyeing of polyamide 6 fabric with new bio-colorant and bio-mordants. Environ. Sci. Pollut. Res..

[78] Islam M.R., Khan A.N.N., Mahmud R.U., Haque S.M.N., Khan M.M.I. (2022). Sustainable dyeing of jute-cotton union fabrics with onion skin (allium CEPA) dye using banana peel (Musa) and guava leaves (*Psidium guajava*) extract as biomordants. Pigment. Resin Technol..

[79] Phromphen P. (2023). Optimization of Marigold Flower Dye Using Banana Peel as a Biomordant. J. Nat. Fibers.

[80] Rather L.J., Shabbir M., Bukhari M.N., Shahid M., Khan M.A., Mohammad F. (2016). Ecological dyeing of woolen yarn with Adhatoda vasica natural dye in the presence of biomordants as an alternative copartner to metal mordants. J. Environ. Chem. Eng..

[81] Madhu A., Chakraborty J.N. (2017). Developments in application of enzymes for textile processing. J. Clean. Prod..

[82] Quandt C., Kuhl B. (2001). Enzymatic processes: Operational possibilities and optimization (Enzymes Possibilite’set perspectives). L’Industrie Text. Issue.

[83] Kumar D., Bhardwaj R., Jassal S., Goyal T., Khullar A., Gupta N. (2021). Application of enzymes for an eco-friendly approach to textile processing. Environ. Sci. Pollut. Res..

[84] Duran N., Duran M. (2000). Enzyme applications in the textile industry. Rev. Prog. Color. Relat. Top..

[85] Vankar P.S., Shanker R., Srivastava J. (2007). Ultrasonic dyeing of cotton fabric with aqueous extract of Eclipta alba. Dye. Pigment..

[86] Zhang Y., Rather L.J., Li Q. (2022). Recent advances in the surface modification strategies to improve functional finishing of cotton with natural colourants—A review. J. Clean. Prod..

[87] Samant L., Jose S., Rose N.M., Shakyawar D.B. (2022). Antimicrobial and UV protection properties of cotton fabric using enzymatic pretreatment and dyeing with Acacia catechu. J. Nat. Fibers.

[88] Benli H., Bahtiyari M.İ. (2015). Use of ultrasound in biopreparation and natural dyeing of cotton fabric in a single bath. Cellulose.

[89] Raja AS M., Thilagavathi G. (2011). Influence of enzyme and mordant treatments on the antimicrobial efficiency of natural dyes on wool materials. Asian J. Text.

[90] Zhang R.P., Cai Z.S. (2011). Study on the natural dyeing of wool modified with enzyme. Fibers Polym..

[91] Islam S., Mohammad F. (2015). High-energy radiation induced sustainable coloration and functional finishing of textile materials. Ind. Eng. Chem. Res..

[92] Adeel S., Gulzar T., Azeem M., Saeed M., Hanif I., Iqbal N. (2017). Appraisal of marigold flower-based lutein as natural colorant for textile dyeing under the influence of gamma radiations. Radiat. Phys. Chem..

[93] Moholkar V.S., Nierstrasz V.A., Warmoeskerken M.M.C.G. (2003). Intensification of mass transfer in wet textile processes by power ultrasound. Autex Res. J..

[94] Czaplicki Z., Matyjas-Zgondek E., Strzelecki S. (2021). Scouring of Sheep Wool Using an Acoustic Ultrasound Wave. Fibres Text. East. Eur..

[95] Peila R., Grande G.A., Giansetti M., Rehman S., Sicardi S., Rovero G. (2015). Washing off intensification of cotton and wool fabrics by ultrasounds. Ultrason. Sonochemistry.

[96] Davulcu A., Eren H.A., Avinc O., Erişmiş B. (2014). Ultrasound assisted biobleaching of cotton. Cellulose.

[97] Kadam V.V., Goud V., Shakyawar D.B. (2013). Ultrasound Scouring of Wool and Its Effects on Fibre Quality.

[98] Al Kashouty M., Elsayad H., Twaffiek S., Salem T., Elhadad S. (2020). An Overview: Textile Surface Modification by Using Sol-gel Technology. Egypt. J. Chem..

[99] Ferrero F., Migliavacca G., Periolatto M. (2016). UV treatments on cotton fibers. Cotton Research.

[100] Adeel S., Bhatti I.A., EL-Nagar K., Alam M.M., Ali N. (2011). Dyeing of cotton fabric using UV irradiated turmeric (*Curcuma longa* L.) as natural dye. Res. J. Text. Appar..

[101] Gulzar T., Adeel S., Hanif I., Rehman F., Hanif R., Zuber M., Akhtar N. (2015). Eco-friendly dyeing of gamma ray induced cotton using natural quercetin extracted from acacia bark (*A. nilotica*). J. Nat. Fibers.

[102] Rehman F., Adeel S., Hanif R., Muneer M., Zia K.M., Zuber M., Khosa M.K. (2017). Modulation of marigold based lutein dye and its dyeing behaviour using UV radiation. J. Nat. Fibers.

[103] Bhatti I.A., Adeel S., Ur Fazal R., Irshad M., Abbas M. (2012). Effect of mercerization and gamma irradiation on the dyeing behaviour of cotton using stilbene based direct dye. Radiat. Phys. Chem..

[104] Ajmal M., Adeel S., Azeem M., Zuber M., Akhtar N., Iqbal N. (2014). Modulation of pomegranate peel colourant characteristics for textile dyeing using high energy radiations. Ind. Crops Prod..

[105] Batool F., Adeel S., Azeem M., Khan A.A., Bhatti I.A., Ghaffar A., Iqbal N. (2013). Gamma radiations induced improvement in dyeing properties and colorfastness of cotton fabrics dyed with chicken gizzard leaves extracts. Radiat. Phys. Chem..

[106] Chirila L., Popescu A., Cutrubinis M., Stanculescu I., Moise V.I. (2018). The influence of gamma irradiation on natural dyeing properties of cotton and flax fabrics. Radiat. Phys. Chem..

[107] Shahidi S., Wiener J. (2016). Radiation effects in textile materials. Radiation Effects in Materials.

[108] Van der Sluijs M.H., Church J.S. (2013). The effect of quarantine-level gamma irradiation on cotton fiber and its subsequent textile processing performance. Text. Res. J..

[109] Khan A.A., Iqbal N., Adeel S., Azeem M., Batool F., Bhatti I.A. (2014). Extraction of natural dye from red calico leaves: Gamma ray assisted improvements in colour strength and fastness properties. Dye. Pigment..

[110] Henniges U., Okubayashi S., Rosenau T., Potthast A. (2012). Irradiation of cellulosic pulps: Understanding its impact on cellulose oxidation. Biomacromol.

[111] Kashiwagi M., Hoshi Y. (2012). Electron beam processing system and its application. SEI Tech. Rev..

[112] Elmaaty T.A., Okubayashi S., Elsisi H., Abouelenin S. (2022). Electron beam irradiation treatment of textiles materials: A review. J. Polym. Res..

[113] Elmaaty T.A., Abouelenin S., Elsisi H., Okubayashi S. (2022). Ecofriendly approach for dyeing synthetic fabrics with natural dyes using electron beam irradiation. Fibers Polym..

[114] Samanta K.K., Basak S., Chattopadhyay S.K. (2014). Environment-friendly textile processing using plasma and UV treatment. Roadmap to Sustainable Textiles and Clothing: Eco-Friendly Raw Materials, Technologies, and Processing Methods.

[115] Vesel A., Mozetic M., Strnad S., Peršin Z., Stana-Kleinschek K., Hauptman N. (2009). Plasma modification of viscose textile. Vacuum.

[116] Molina R., Teixidó J.M., Kan C.W., Jovančić P. (2017). Hydrophobic coatings on cotton obtained by in situ plasma polymerization of a fluorinated monomer in ethanol solutions. ACS Appl. Mater. Interfaces.

[117] Okuno T.Y.H.Y.T., Yasuda T., Yasuda H. (1992). Effect of crystallinity of PET and nylon 66 fibers on plasma etching and dyeability characteristics. Text. Res. J..

[118] Shah J., Shah S. (2013). Innovative plasma technology in textile processing: A step towards a green environment. Res. J. Eng. Sci.

[119] Haji A. (2019). Dyeing of cotton fabric with natural dyes improved by mordants and plasma treatment. Prog. Color Color. Coat..

[120] Haji A., Qavamnia S.S., Bizhaem F.K. (2016). Optimization of oxygen plasma treatment to improve the dyeing of wool with grape leaves. Ind. Textila.

[121] Ahmed N.S.E., El-Shishtawy R.M. (2010). The use of new technologies in coloration of textile fibers. J. Mater. Sci..

[122] Baig U., Khatri A., Ali S., Sanbhal N., Ishaque F., Junejo N. (2021). Ultrasound-assisted dyeing of cotton fabric with natural dye extracted from marigold flower. J. Text. Inst..

[123] Babar A.A., Peerzada M.H., Naveed T., Dayo A.Q. (2019). Exhaust reactive dyeing of lyocell fabric with ultrasonic energy. Ultrason. Sonochem..

[124] Udrescu C., Ferrero F., Periolatto M. (2014). Ultrasound-assisted dyeing of cellulose acetate. Ultrason. Sonochem..

[125] Xue Z. (2016). Study of dyeing properties of wool fabrics treated with microwave. J. Text. Inst..

[126] Arain R.A., Ahmad F., Khatri Z., Peerzada M.H. (2021). Microwave assisted henna organic dyeing of polyester fabric: A green, economical and energy proficient substitute. Nat. Prod. Res..

[127] Van Der Kraan M. (2007). Equilibrium study on the disperse dyeing of polyester textile in supercritical carbon dioxide. Text. Res. J..

[128] Zheng H., Zhang J., Yan J., Zheng L. (2016). An industrial scale multiple supercritical carbon dioxide apparatus and its eco-friendly dyeing production. J. CO2 Util..

[129] Penthala R., Kumar R.S., Heo G., Kim H., Lee I.Y., Ko E.H., Son Y.A. (2019). Synthesis and efficient dyeing of anthraquinone derivatives on polyester fabric with supercritical carbon dioxide. Dye. Pigment..

[130] Eren H.A., Yiğit İ., Eren S., Avinc O. (2020). Sustainable textile processing with zero water utilization using supercritical carbon dioxide technology. Sustainability in the Textile and Apparel Industries: Production Process Sustainability.

[131] Banchero M. (2020). Recent advances in supercritical fluid dyeing. Color. Technol..

[132] Meksi N., Moussa A. (2017). A review of progress in the ecological application of ionic liquids in textile processes. J. Clean. Prod..

[133] Ragheb A.A., Tawfik S., Abd-El Thalouth J.I., Mosaad M.M. (2017). Development of printing natural fabrics with curcuma natural dye via nanotechnology. Int. J. Pharm. Sci. Res..

[134] Ragheb A., Mosaad M., Mahmoud S., Abd Thaloth J.I. (2019). The Impact of Nanotechnologies on developing the printing of natural fabrics with pomegranate peel. Egypt. J. Chem..

[135] Wasserscheid P., Welton T. (2018). Ionic Liquids in Synthesis.

[136] Lei Z., Chen B., Koo Y.M., MacFarlane D.R. (2017). Introduction: Ionic liquids. Chem. Rev..

[137] Lebeau J.L., Venkatachalam M., Fouillaud M., Dufossé L., Caro Y. Extraction of fungal polyketide pigments using ionic liquids. Proceedings of the 8th International Conference of Pigments in Food, Coloured Foods for Health Benefits.

[138] Bosiljkov T., Dujmic F., Bubalo M.C., Hribar J., Vidrih R., Brncic M., Zlatic E., Redovnikovic I.R., Jokic S. (2017). Natural deep eutectic solvents and ultrasound-assisted extraction: Green approaches for extraction of wine lees anthocyanins. Food Bioprod. Process.

[139] Wang Y., Jin G., Guo B., Zhou Y. (2016). Study on the ionic liquid-based extraction technology of total flavonoids from Broussonetia papyrifera leave. Chem. Ind. Eng. Process.

[140] Mketo N., Nomngongo P.N. (2023). Application of ionic liquids for extraction of phenolic compounds and dyes: A critical review. Green Sustainable Process for Chemical and Environmental Engineering and Science.

[141] Bianchini R., Cevasco G., Chiappe C., Pomelli C.S., Rodríguez Douton M.J. (2015). Ionic liquids can significantly improve textile dyeing: An innovative application assuring economic and environmental benefits. ACS Sustain. Chem. Eng..

[142] Andrade R.S., Torres D., Ribeiro F.R., Chiari-Andréo B.G., Oshiro Junior J.A., Iglesias M. (2017). Sustainable cotton dyeing in nonaqueous medium applying protic ionic liquids. ACS Sustain. Chem. Eng..

[143] Opwis K., Benken R., Knittel D., Gutmann J.S. (2017). Dyeing of PET fibers in ionic liquids. Int. J. New Technol. Res..

